# Patterning and Morphogenesis From Cells to Organisms: Progress, Common Principles and New Challenges

**DOI:** 10.3389/fcell.2020.602483

**Published:** 2020-11-06

**Authors:** Andrew B. Goryachev, Moisés Mallo

**Affiliations:** ^1^SynthSys, Centre for Synthetic and Systems Biology, University of Edinburgh, Edinburgh, United Kingdom; ^2^Gulbenkian Institute of Science (IGC), Oeiras, Portugal

**Keywords:** cell polarity, tissue polarity, morphogenesis, patterning, mechanical forces, phase separation

The ability to progress from simple to more complex, organized, and spatially differentiated forms, or morphogenesis, is, perhaps, one of the most fundamental properties of biological systems from individual cells to large multicellular organisms, to whole populations. Customarily, cell biology is concerned with the morphogenesis of individual cells, while developmental biology studies morphogenesis on the scales of tissues and whole organisms. We envision that the Section “Patterning and Morphogenesis” will take on the challenge of unifying these efforts focusing on their integration based on the common organizing principles. Historically, patterning and morphogenesis has been a fundamentally multidisciplinary area of research that experienced influences of many scientific disciplines outside of biology, i.e., physics, chemistry, and mathematics. Therefore, one of our goals will be to foster this spirit of multidisciplinarity and encourage contributions from classical experimental research as well as from more quantitative and theoretical fields. Even when the development of tools allowing appropriate experimental approaches to complex cellular and molecular activity was still in its infancy, theoretical models were already proposed to account for the formation of complex and highly specific morphological features. Many influential ideas generated by the twentieth century giants, such as Alan Turing, Conrad Waddington, and Lewis Wolpert, have become even more exciting now, when we can actually observe and experimentally perturb their manifestations in living biological systems.

## Seeing is Believing

Currently we experience an unprecedent boom in several areas of technology that have dramatically increased the depth and detail with which the mechanisms of patterning and morphogenetic processes are explored. Most often these technological developments happened independently from each other, but their clever and creative combination has potentiated their individual power, as well as expanded their applicability. Advances in optical microscopy are among the technological developments with the highest impact in the depth, resolution and precision that can be applied to the analysis of a wide variety of cell and molecular behaviors guiding morphogenetic processes in many different *in vivo* and *in vitro* systems. Full exploitation of the power of advanced microscopic technology resulted from its association with other technological areas. For instance, concomitant development of methods to introduce different types of fluorescent labels (Shcherbakova et al., 2014) into cell structural components, many of them compatible with live imaging analyses in cells or even whole organisms, has enabled observation of morphogenetic processes at unprecedented space and time resolution. The use of advanced computational techniques has also significantly increased image analysis power. The recent success of the super resolution microscopy (Sengupta et al., 2012; Betzig, 2015) and the generation of precise active 3D models (Long et al., 2012) are just two examples illustrating the benefits of merging imaging and computation. These improvements allowed live observation of highly dynamic processes, such as establishment of cellular polarity, complex oscillations, and propagation of waves inside cells and tissues (Bement et al., 2015; Tsiairis and Aulehla, 2016; Wu et al., 2018; Landin Malt et al., 2019). This also allowed us to follow cell routes and fates in highly complex structures like a developing mouse embryo (Mcdole et al., 2018). Surely, these are only the appetizers for what we will witness in the coming years.

Efficient single cell technologies have also been introduced in the analysis of a variety of complex morphogenetic processes (Wagner et al., 2018; Cao et al., 2019; Delile et al., 2019; Pijuan-Sala et al., 2019). It is expected that the power of this analytic approach will be considerably increased with the combination of protocols allowing spatial allocation of the individual cells within the tissue of origin, as well as by the assistance of systems biology methodology to infer and model gene networks regulating those processes, also leading to the generation of experimentally testable hypotheses (Tam and Ho, 2020). In addition to analytical approaches, in recent years new technologies have also expanded the toolbox permitting introduction of precisely controlled modifications in the experimental system required to evaluate the role of specific features in the generation of complex structures. The appearance of the CRISPR/Cas9-based genomic editing techniques (Adli, 2018) is among the most relevant of those new approaches, with an immediate deep impact in the field of cell and developmental biology. For instance, it has democratized the use of genetics to the analysis of patterning and morphogenetic processes. Until very recently controlled experimental alterations of gene activity were possible in only a handful of model systems, and even in them the options to modify gene expression were often limited, mostly relying on the introduction of different types of exogenous elements in the genome or interfering with gene expression machinery. CRISPR/Cas9 technology provided for the first time the possibility of introducing reverse genetic approaches to the study of cellular and developmental processes in organisms previously considered unsuited to controlled genetic modification (Martin et al., 2016; Mazo-Vargas et al., 2017; Rasys et al., 2019).

Another group of technological advances relates to the introduction of new *in vitro* model systems closely simulating *in vivo* conditions that for a variety of reasons cannot be effectively approached in their natural environment. To name but a few, *in vitro* reconstitutions of the MinCDE cell patterning system (Loose et al., 2008; Glock et al., 2019), mixtures of cytoskeletal polymers and their cognate molecular motors (Koenderink et al., 2009; Opathalage et al., 2019), and whole mimetic actomyosin cortices (Carvalho et al., 2013; Foster et al., 2019), dramatically accelerated our understanding of the morphogenesis in the corresponding *in vivo* systems. Some of these *in vitro* approaches had been around already for a number of years. Indeed, embryoid bodies, Keller sandwiches and a variety of tissue explants represent three classical examples of such systems that have been effectively used for decades (Doetschman et al., 1985; Keller and Danilchik, 1988; Freshney, 2016). However, the technologies for the generation of complex *in vitro* models evolved considerably from these early systems and are now mostly represented by structures globally known as organoids (Kretzschmar and Clevers, 2016), when they aim at mimicking specific organ structures like the brain or the intestine, and gastruloids (Beccari et al., 2018), when they intend to reproduce early stages in embryonic development.

## Quo Vadis?

Given these fascinating technological advances, what are the main challenges that the broad field of patterning and morphogenesis is currently facing? Owing to the impact of the concept of morphogens introduced by Turing (1952), chemical regulation of morphogenesis has been in the spotlight for decades. Indeed, the concept of morphogens further developed by Wolpert and colleagues turned out to be exceptionally fruitful (Smith et al., 2008; Green and Sharpe, 2015; Wolpert, 2016). Supported by the discussed above modern technologies, we are presently confident that many proteins, e.g., TGFβ, bone morphogenetic proteins, sonic hedgehog and WNTs, fully qualify for the role of extracellular morphogens. Interestingly, the concept of morphogens can be also productively extended into intracellular morphogenesis within the framework of the activator-substrate model (Hubatsch and Goehring, 2020). Perhaps, the best characterized intracellular morphogens are small GTPases that form membrane localized prepatterns for cytoskeletal structures and in the context of the establishment of cell polarity (Goryachev and Leda, 2017, 2019, 2020). The concept of mechanical regulation of morphogenesis was formed a long time ago, perhaps even before that of chemical regulation. However, due to the experimental difficulties and the paucity of theoretical approaches it remained in the shadow of the chemical regulation. This situation changed dramatically in the past decade due to the burgeoning development of experimental and theoretical biomechanics. One of the current grand challenges is to integrate chemical and mechanical mechanisms to achieve a new level in understanding of morphogenesis on both intracellular and multicellular levels (Howard et al., 2011; Goehring and Grill, 2013; Gross et al., 2017).

Arguably, the most recent development in the mechanisms of morphogenesis has been the recognition of the role played by the phenomenon of phase separation (Hyman et al., 2014; Banani et al., 2017). Phase separation is a well-developed concept in physics and chemistry but its relevance to biology in general and morphogenesis in particular has been largely unexplored. Presently evidence is abound that phase separation is the governing principle in control of formation of multiple cellular organelles not bound by a lipid membrane, such as centrosomes, nucleolus, and a large variety of cytoplasmic and nuclear granules and bodies (Woodruff et al., 2018; Alberti et al., 2019). The out of equilibrium nature of biological phase separation makes all the difference as it converts a rigid unidirectional aggregation of molecules into a highly plastic process which is finely controlled by cellular signaling. Another current grand challenge is to understand these mechanisms of regulation and to integrate them with the better understood patterning mechanisms by reaction and diffusion. While currently phase separation appears mainly relevant to the intracellular morphogenesis, it is not unlikely that phase separation also plays roles on the multicellular scale, e.g., in the formation of basal membranes, and even tissue and organism scales, such as in fascia and cartilage.

It is exciting to anticipate what the development of these new conceptual and technical advances will teach us about how cells work and interact to build complex functional structures. We hope that, in the years to come, the Patterning and Morphogenesis Section of the Frontiers in Cell and Developmental Biology will play an important role in disseminating this new knowledge among the scientific community and beyond.

## Author Contributions

All authors listed have made a substantial, direct and intellectual contribution to the work, and approved it for publication.

## Conflict of Interest

The authors declare that the research was conducted in the absence of any commercial or financial relationships that could be construed as a potential conflict of interest.
